# Incidental finding of a bilateral complete ossification of stylo‐hyoid chain and thyro‐hyoid ligaments

**DOI:** 10.1002/ccr3.5789

**Published:** 2022-04-27

**Authors:** Eya Moussaoui, Sahar Kadri, Lamia Oualha, Nabiha Douki

**Affiliations:** ^1^ 37966 Department of dental medicine SAHLOUL Hospital (Sousse) Dental Faculty of Monastir University of Monastir Sousse Tunisia; ^2^ 37966 Laboratory of oral health and maxillofacial rehabilitation (LR12ES11) University of Monastir Sousse Tunisia

**Keywords:** eagle syndrome, pseudarthroses, stylohyoid ligament, styloid chain, tyro‐hyoid ligament

## Abstract

Ossification of stylohyoid chain corresponds to the ossification of stylohyoid ligament that can vary from thin short to thick long ossification and can be associated with other calcifications. We report in this case a bilateral non painful complete ossification of the stylohyoid complex extended to the thyrohyoidien ligament.

## Graphical abstract

Ossification and elongation of the stylohyoid chain can cause neck and facial symptoms, known as Eagle's syndrome. Nonetheless, a bilateral ossified and elongated stylohyoid chain associated with ossification of the thyro‐hyoid ligament may be not painful.

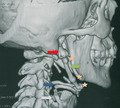

A 53‐year‐old man consulted the dental medicine department for a dental check‐up. A routine conventional dental radiographic examination was performed and showed bilateral ossification of the stylohyoid complex (Figure 1), then confirmed by a lateral neck X‐ray (Figure 2) that showed ossification of the styloid chain (styloid Process, stylohyoid ligament, and lesser cornua of the hyoid bone) extended to the thyrohyoidien ligament. The patient had no pain, with very restricted head and neck movements.

**FIGURE 1:**
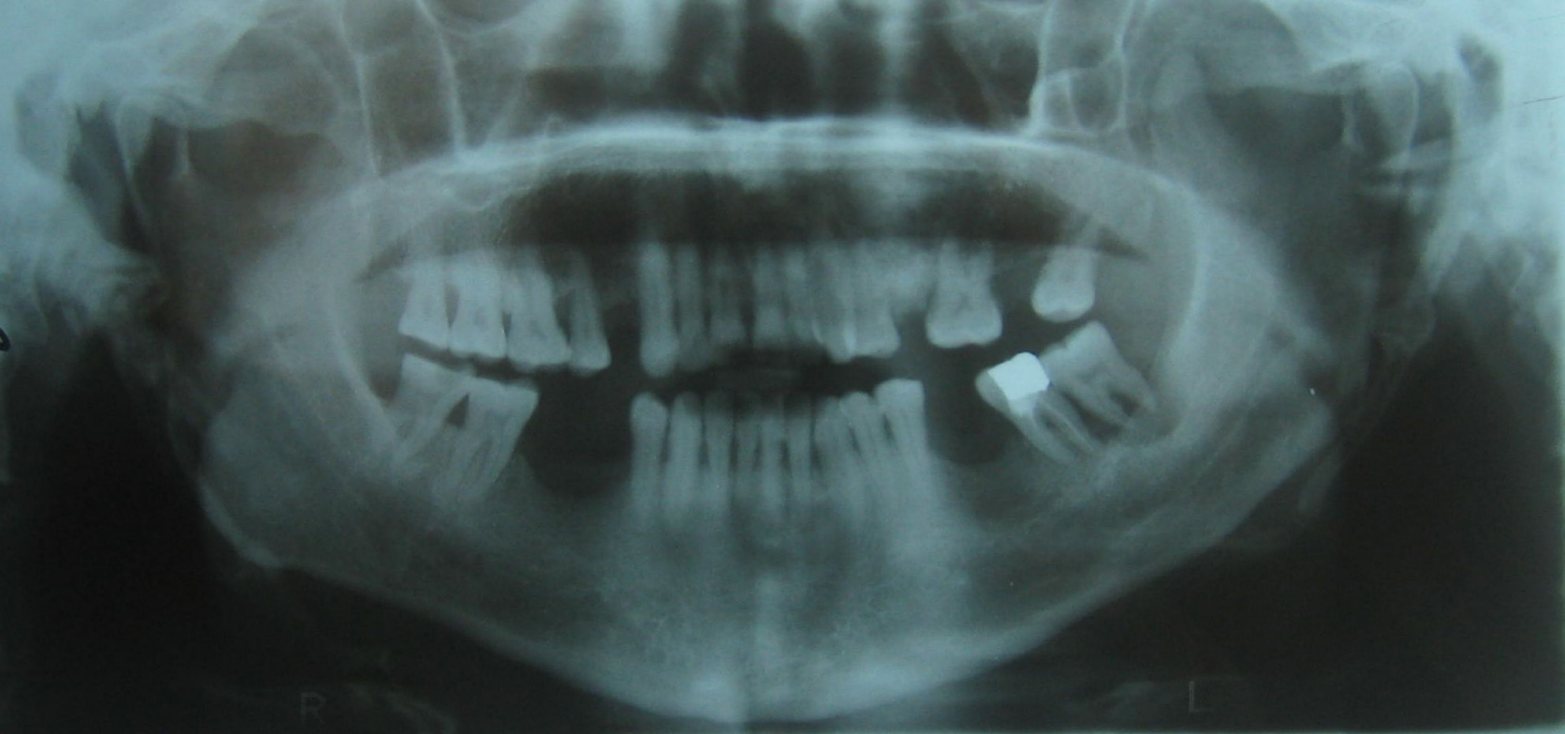
bilateral ossification of the stylohyoid complex visualized in the panoramic X‐ray

**FIGURE 2:**
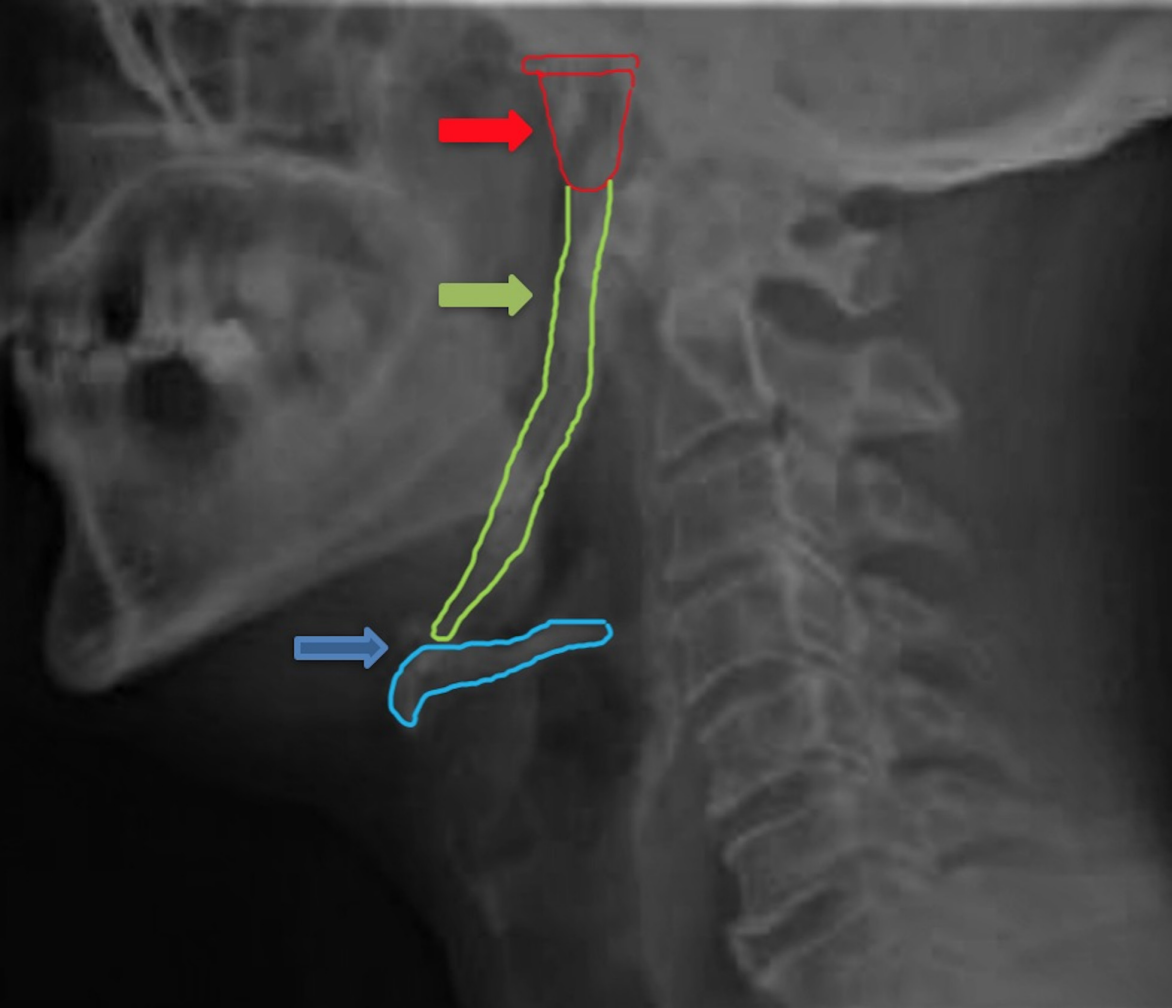
Lateral view of neck demonstrating styloid chain ossification. 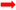
Styloid process of the temporal bone, 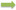
Ossification of stylohyoid ligament, 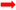
Hyoid bone

He reported the history of severe cervical trauma caused by a fall down stairs 30 years ago.

CT scans of the neck showed an elongated and heavily ossified styloid process, the stylohyoidien ligament extending to the lesser cornua of the hyoid bone with three pseudarthroses, associated with a bilateral ossification of the thyrohyoid ligaments (Figure 3).

**FIGURE 3:**
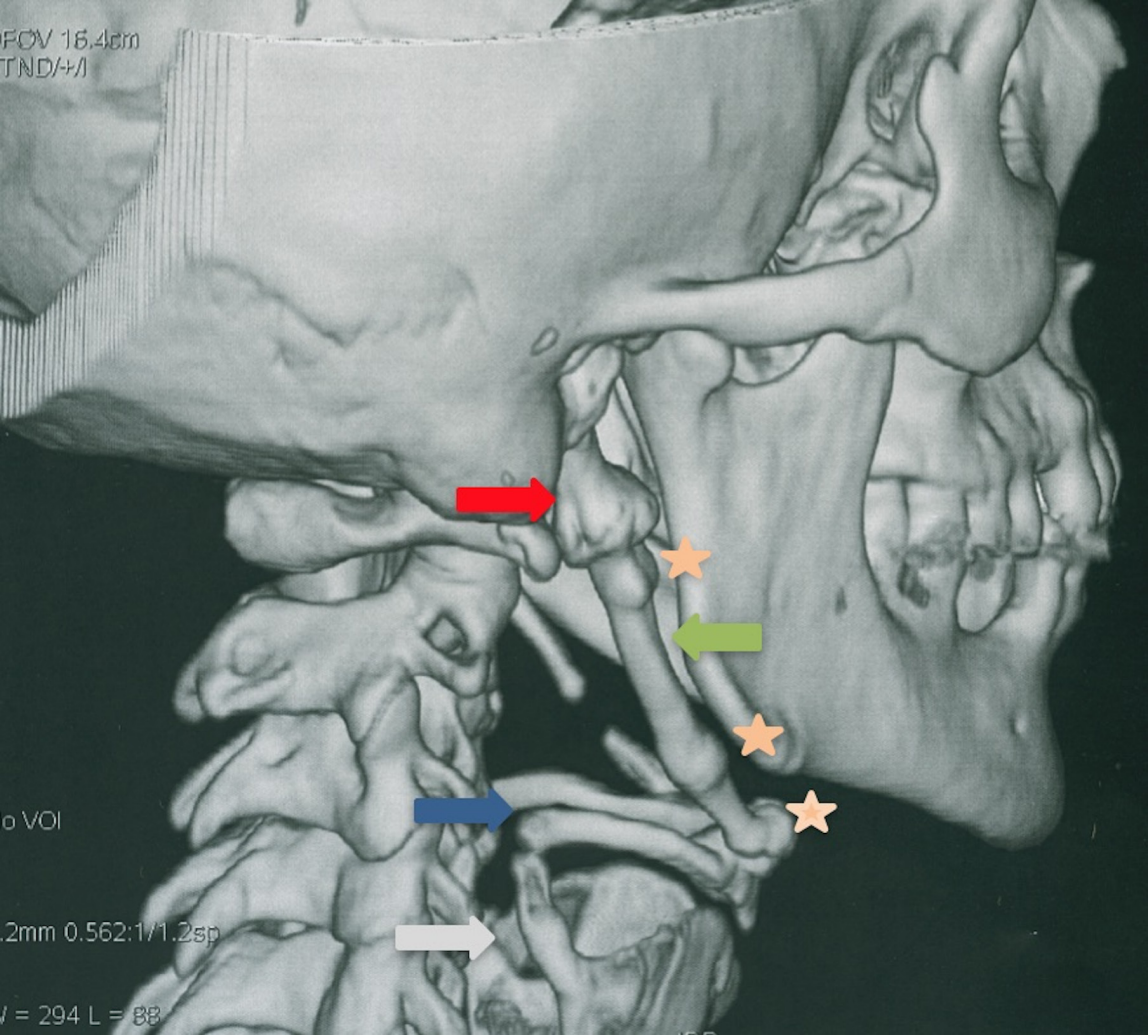
CT scan (3D VRT) of the neck shows complete ossified styloid chain and thyrohyoid ligament. 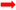
Styloid process, 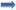
Hyoid bone, 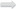
Ossification of thyrohyoid ligament, 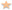
Pseudoarthroses, Ossification of stylohyoid ligament

The stylohyoid complex (SHC) consists of the styloid process, the stylohoid ligament, and the lesser cornu of the hyoid bone.^2^ Its mineralization cause usually oro‐facial pain defining the Eagle's syndrome^1^, ^2^; however, it can be non‐painful and discovered incidentally as in our case. A previous cervical trauma or surgery has been frequently reported as the main cause.^2^ In absence of symptomatology, no treatment is required.^2^ The case reported here is specific because of the non‐painful bilateral ossified stylohyoid chain that is associated with ossification of the thyro‐hyoid ligament. Despite this thick and long calcification, no surgical treatment was needed.

## CONFLICTS OF INTEREST

All the authors declare that there is no conflicts of interest.

## AUTHOR CONTRIBUTIONS

Eya moussaoui performed data collection and interpretation and drafting the manuscript. Sahar kadri involved in collection and interpretation of data. Ouaha lamia revising the manuscript for important intellectual content. Nabiha douki performed conception and design and revising the manuscript.

## ETHICAL APPROVAL

Data from the patient included in this case report were treated anonymously, and a statement of informed consent was signed to allow the use of her medical and dental records and photos.

## CONSENT

Written patient consent has been signed and collected in accordance with the journal's patient consent policy.

## Data Availability

Data openly available in a public repository that issues datasets with DOIs
